# Subclinical Iodine Deficiency among Pregnant Women in Haramaya District, Eastern Ethiopia: A Community-Based Study

**DOI:** 10.1155/2014/878926

**Published:** 2014-07-17

**Authors:** Haji Kedir, Yemane Berhane, Alemayehu Worku

**Affiliations:** ^1^College of Health and Medical Sciences, Haramaya University, P.O. Box 235, Harar, Ethiopia; ^2^Addis Continental Institute of Public Health, Addis Ababa, Ethiopia; ^3^School of Public Health, Addis Ababa University, P.O. Box 26751/1000, Addis Ababa, Ethiopia

## Abstract

*Background.* Iodine deficiency in pregnancy is a worldwide problem. This study aimed to assess prevalence and predictors of subclinical iodine deficiency among pregnant women in Haramaya district, eastern Ethiopia. *Methods.* A cross-sectional, community-based study was conducted on 435 pregnant women existing in ten randomly selected rural kebeles (kebele is the smallest administrative unit in Ethiopia). Data on the study subjects' background characteristics, dietary habits, and gynecological/obstetric histories were collected via a structured questionnaire. UIC of <150 *μ*g/L defined subclinical iodine deficiency. Data were analyzed by Stata 11. A multivariable logistic regression was used to identify the predictors of subclinical iodine deficiency. *Results.* The median urinary iodine concentration (MUIC) was 58.1 *μ*g/L and 82.8% of the women who had subclinical iodine deficiency. The risk of subclinical iodine deficiency was reduced by the use of iodized salt (AOR = 0.13) and by intake of milk twice a month or more (AOR = 0.50), but it was increased by maternal illiteracy (AOR = 3.52). *Conclusion.* Iodine nutritional status of the pregnant women was poor. This shows that women and their children are exposed to iodine deficiency and its adverse effects. Thus, they need urgent supplementation with iodine and improved access to and intake of iodized salt and milk during pregnancy.

## 1. Background

Iodine is an essential nutrient which is used in the synthesis of thyroid hormones that are important for the normal development of the nervous system [1]. Its inadequate intake brings iodine deficiency disorders (IDD) and hypothyroidism, either clinical or subclinical, is one of them [2]. Previous researchers have reported that during pregnancy, mild iodine deficiency causes maternal hypothyroxinemia that is related to mild and subclinical cognitive and psychomotor deficit in neonates, infants, and children [3]. Several others have shown that severe iodine deficiency during pregnancy increases the risk of spontaneous abortion, stillbirth, reduced intelligence, neurological cretinism, poor cognitive functions, and delayed psychomotor developments [1, 2, 4, 5].

Nearly two billion (28%) of the world's population, of whom more than 321 million (39%) are Africans, are at risk of insufficient iodine intake [6]. Based on the global data of iodine nutrition, Ethiopia is categorized among moderately iodine deficient countries [6]. Similarly, Ethiopia is one of the African countries with the highest prevalence of IDD and with the weakest program to prevent IDD [7]. From 34.5% to 37% of the childbearing women in the country have goiter [8].

Because of the higher production and fetal transfer of maternal thyroid hormone, fetal iodine transfer, and raised renal iodine clearance [9], pregnant women need more iodine than their counterparts do. They are required to consume 250 *μ*g of iodine a day [10]. However, they hardly meet their requirement solely from the usual diets [10, 11].

Universal salt iodization (USI) is the most cost effective strategy recommended to eliminate IDD [12, 13]. However, more than one-third of the African households do not have access to iodized salt [14]. Only 20% of the Ethiopian households use adequately iodized salt [15]. Thus, iodine deficiency has been identified as a serious public health problem for the Ethiopian population. As a result a national regulatory provision for mandatory universal salt iodization was offered in 2011.

Still, hardly any of the previous studies done on the problem in Ethiopia have considered subclinical iodine deficiency in pregnant women. Therefore, this study aimed to determine median urinary iodine concentration, prevalence of subclinical iodine deficiency, and factors associated with iodine deficiency among the pregnant women in rural communities of Haramaya district of eastern Ethiopia.

## 2. Methods 

### 2.1. Study Setting, Design, and Period

A community-based cross-sectional study was done in Haramaya district, eastern Ethiopia, from March 16 to 29, 2012. The district is 1400–2340 meters above sea level. It is divided into 4 semiurban kebeles and 33 rural kebeles (the lowest administrative units) and has a population of about 271,018 people, of whom more than 96% are Oromo in ethnicity and Muslim. We have described the study area in more detail in the previously published article [16].

### 2.2. Study Population, Sample Size, and Sampling

Pregnant women in the randomly selected rural kebeles of the district were the study population. Pregnant women were identified by self-report, from the report of health extension workers in the kebeles and urine testing when pregnancy was doubtful. The sample size was estimated by assuming a 50% subclinical iodine deficiency, a 95% confidence interval, a 5% margin of error, and a 15% nonresponse rate and this yielded a sample size of 443. According to Andersen et al., a sample size of 500 subjects is adequate to assess iodine nutrition of the population from spot samples [17].

This study is a subsample of a prospective cohort study of the effects of maternal nutrition on birth outcomes. It was based on random subsample of ten of the twelve kebeles selected for the cohort study. To select the kebeles (primary sampling units), all the rural kebeles in the district (*n* = 33) were listed and each was assigned a unique number. Then, a simple random sampling technique was applied to select the twelve kebeles for the cohort study. Finally, the list of the twelve kebeles was considered as a sampling frame and ten kebeles were randomly selected from it. Cognizing the little difference between the larger estimated sample size (*n* = 500) and the number of the pregnant women in the ten selected kebeles (*n* = 525), we recruited all of them.

### 2.3. Data Collection Methods and Procedures

Eight college graduates collected the data and two public health professionals and the principal investigator supervised the fieldwork. The respondents' sociodemographic, reproductive history and dietary characteristics were obtained via a pretested and interviewer-administered questionnaire. The items included in the questionnaire were taken from the Ethiopian Health and Demographic Survey (EDHS) and were adapted to suit the study context.

A teaspoon of salt sample was taken from each household of the respondent and tested for contents of iodine, using a rapid test kit distributed by UNICEF for the purpose of assessing household salt iodine content. The rapid test kit comprises test solution and a color chart. We put a drop of the test solution on each salt sample. Then, salt samples that immediately turned into a purple blue color of any intensity as shown on the color chart after putting a drop of test solution were classified as containing iodine and those not remaining unchanged after putting a drop of test solution were classified as not containing iodine.

A 10 mL urine sample was taken from each study subject in wide-opened plastic caps covered by an opaque paper bag and transferred into a labeled, clean tightly sealed plastic tubes that were free from iodine or any other chemical to avoid leakage and cross-contaminations with iodine from other sources. The urine samples were kept in a cold box and transported to iodine laboratory at Ethiopian Health and Nutrition Research Institute (EHNRI) under the cold chain. In the national laboratory of food and nutrition research at ENHRI, duplicates of each urine sample brought from each pregnant women were prepared and the determination of urinary iodine concentration was made using the duplicate samples. The Sandell-Kolthoff reaction method which is recommended by World Health Organization (WHO), United Nations Children's Fund (UNICEF), and International Council for the Control of Iodine Deficiency Disorders (ICCIDD) was used to determine the UIC [18]. The method is described in further detail by the publication of WHO [18].

### 2.4. Statistical Analysis

Data were double-entered and validated by EpiData Version 3.1. Stata 11 and SPSS V. 16 were used to analyze the data. Using Mann-Whitney *U* test or Kruskal-Wallis test, we compared the median and the UIC between groups of categorical independent variables. Through bivariate and multivariable logistic regressions, the predictors of subclinical iodine deficiency (UIC <150 *μ*g/L) were identified. Variables were included into the multivariable model based on the existing literature about their suspected effects on subclinical iodine deficiency and its predictors. In order to avoid confounding factors, the enter method of logistic regression was used and the risk estimates were adjusted for all the variables entered. A two-sided *P* value of <0.05 was considered to declare statistical significance.

WHO recommends urinary iodine concentrations of <150, 150–249, 250–499, and ≥500 *μ*g/L that should be used to indicate insufficient, adequate, more than adequate, and excessive levels of iodine intake in population of pregnant women [19]. In this study, UIC <150 *μ*g/L defined subclinical iodine deficiency and value ≥150 *μ*g/L absence of subclinical iodine deficiency. Median UIC and subclinical iodine deficiency were the dependent variables. The group with <150 *μ*g/L were further categorized into <20 *μ*g/L (severe iodine deficiency), 20–49 *μ*g/L (moderate iodine deficiency), and 50–149 *μ*g/L (mild iodine deficiency) [20]. The independent variables and covariates examined for their association with subclinical iodine deficiency were maternal age, educational status, family size, possession of milk cows, consumption of milk, consumption of cabbage, household use of iodized salt, prenatal visit, trimester of pregnancy, and number of pregnancies.

### 2.5. Ethics

The Institutional Review Board (IRB) of Haramaya University and the National Research Ethics Review Committee of Ethiopia reviewed and approved the protocol. All the study participants provided their written informed consent. The purposes, the data collection procedures, the risks, and the benefits of the research were explained to the eligible respondents before obtaining their informed consent.

## 3. Results

### 3.1. Sociodemographic Characteristics

Of the 525 pregnant women recruited, 435 were included in the analysis and the response rate was 82.8%. The remaining 90 pregnant women did not give their urine and they were not different from the women included in the analysis in their sociodemographic characteristics. Nearly all of the respondents were Oromo (99.5%) and Muslim (99.3%). Their mean age and duration of pregnancy were 27.0 (SD ± 5.9) years and 25.8 (SD ± 5.8) weeks, respectively. The characteristics of women participating in this study are detailed in Table 1.

*Urinary Iodine Concentration and Associated Factors.* The median UIC was 58.1 *μ*g/L (interquartile range (IQR) = 21.4–111.1). The women in the households which used iodized salt more than those in the households that did not and women who consumed milk at least twice a month more than those who never did so had significantly higher median UIC (Mann-Whitney *U* test, *P* < 0.05) (Table 2). However, the median UIC did not show significant associations with the respondents' educational status, parity levels, trimester of pregnancy, and other sociodemographic characteristics studied (Table 2).

*Subclinical Iodine Deficiency and Its Predictors.* In this study, 360 (82.8%) respondents had subclinical iodine deficiency. Of the total respondents, 40 (9.2%), 35 (8.0%), and none had adequate, more than adequate, and excessive iodine intakes, respectively. When the levels of subclinical iodine deficiency were further categorized, 105 (24.1%), 91 (20.9%), and 164 (37.7%) of all respondents had UIC of <20 *μ*g/L (severe iodine deficiency), 20–49 *μ*g/L (moderate iodine deficiency), and 50–149 *μ*g/L (mild iodine deficiency), respectively.

In the bivariate analysis, the risk of subclinical iodine deficiency was significantly low among the women who were 35 to 49 years of age, who consumed milk at least three times a month, who eat cabbage once a week or more frequently, and who were from households which used iodized salt (Table 3).

In a multivariable analysis, the factors significantly associated with subclinical iodine deficiency at the bivariate still remained significant. Thus, the respondents who used iodized salt had 87% lower risk of subclinical iodine deficiency than their counterparts who did not and those who consumed milk at least twice a month had a 50% lower risk of the deficiency (Table 3). The risk of subclinical iodine deficiency increased by more than threefold in illiterate women than literate ones and in women within a family size of five or more than in those within family size below five, while the magnitude of subclinical iodine deficiency was significantly reduced with increased parity level (Table 3).

## 4. Discussion

The study showed a high prevalence of subclinical iodine deficiency, a low median UIC, and a low proportion of households with iodized salt, while consuming iodized salt and milk that were predictors of higher UIC and were associated with reduced prevalence of subclinical iodine deficiency. Lack of education among pregnant women was associated with a threefold increased risk of subclinical iodine deficiency.

Recently, other researchers working in the Sidama Zone of southern Ethiopia on 172 pregnant women have witnessed that iodized salt is not used in higher than 90% of the households and 60% of the respondents had UIC lower than 20 *μ*g/L and the median UIC of 15 *μ*g/L [21]. Compared to the study from southern Ethiopia, the present study observed lower proportion of pregnant women with UIC less than 20 *μ*g/L and a higher median UIC [21]. Nevertheless, both studies indicate that iodine deficiency is unacceptably high in Ethiopian pregnant women. The differences between the findings of our study conducted in eastern Ethiopia and the study from the southern Ethiopia could be explained by sociocultural and ecological differences and the compositions of the study subjects enrolled in the two studies. The variations could also indicate the need of regional studies with detailed investigation of risk factors specific to particular locality or a nationwide study with representative coverage of different regions and contextual risk factors.

High level of iodine deficiency has been witnessed by previous clinical and subclinical assessments made on samples of school-aged children [22, 23]. For example, a median UIC of 56 *μ*g/L and 34.2 *μ*g/L is reported from southwestern and southern Ethiopia, respectively [22, 23]. Despite the differences in the study subjects, the findings from the present study were confirmations of the degree of iodine deficiency in Ethiopia.

Like other authors, we observed that household salt iodization significantly reduces the risk of iodine deficiency [24, 25]. However, low proportion of iodized salt utilization at the household level reported in our study calls for urgent need of coordinated efforts. Our study and other previous studies demonstrated that milk is an important source of iodine [25, 26], and this strengthens the promotion of the intake of milk and other dairy products during or before prenatal period and of the importance of possessing milk cows at the household level in the communities like ours that do not have ease of access to fish and other sea foods. In our study, the illiterate study subjects were more likely to be iodine deficient than the literate ones. It has also been demonstrated by other authors that better educated pregnant women are more likely to consume food with higher dietary quality index [27]. The decrease of the UIC in advanced stages of pregnancy that have been demonstrated by several others [28–30] was not confirmed by the present study. This could be due to the small number of respondents with the first trimester of pregnancy in the present study.

Unlike the understanding of the negative effect of repeated pregnancy on maternal nutritional status, in this study the risk of subclinical iodine deficiency decreased with an increase in the number of pregnancy experiences. This could be explained by the possibility that pregnant women with more pregnancy experiences could receive nutrition related information from various sources during their previous pregnancy experience and as a result they may have higher iodine intake [31].

Our study might have had some limitations. The questionnaire based assessment of dietary iodine intake and other data might not be as accurate as it should be. Moreover, as UIC can only indicate the present iodine nutrition, the findings might not reflect levels of chronic iodine deficiency. To get an insight on long standing iodine deficiency, it would have been better if the prevalence of goiter had also been assessed. Furthermore, UIC data cannot tell thyroid function in the group of women studied, which could only be possible with determination of thyroid hormones. The data on thyroid hormones and prevalence of goiter were not possible to obtain with the resource available for the present study. Nevertheless, the data are important for better understanding of the iodine deficiency in this community. Another limitation is that this study is a cross-sectional study conducted during the hot season which might lead to the underestimation of iodine deficiency and overestimation of median urinary iodine concentration as a result of seasonal variation in urinary iodine concentration that increases during the dry hot season [32]. In addition, the household salt iodization test was qualitative; it did not provide iodine concentration. Even though it has been recommended that salt samples identified as containing iodine based on the color change should be further examined through iodiometric titration for the quantification of iodine content and evaluation of its adequacy [18], this was not done in the present study, because only a few samples were found to contain iodine based on the rapid test and further classification of such a small number would make further analysis impossible. Had it not been for the resource limitation, we could have considered design effect to account for the cluster based sampling. However, the effect of this problem could be minimal due to the inclusion of all pregnant women that were identified in each selected kebele.

Regardless of its limitations, the present study contributed to the global body of knowledge by establishing the Ethiopian data on UIC and iodine nutrition among pregnant women. It is a contribution of Ethiopian context to the global body of knowledge regarding iodine deficiency during pregnancy [33, 34].

This study also revealed high prevalence of subclinical iodine deficiency and the scarcity of iodized salt usage among the households in eastern Ethiopia using the two simple and practical methods recommended [35]. Therefore, the findings have implications for public policy, health service provision, self-care, and future research work.

The legislation of Universal Salt Iodization (USI) law is an indication of political commitment and is a corner stone in the prevention of iodine deficiency, but it may not be adequate enough as a self-standing strategy to meet the goal. A number of previous studies have reported persistent occurrence of IDD in pocket geographical locations of countries, regardless of the USI law in place [36]. The same authors witnessed that public awareness about the importance of iodized salt and appropriate handlings of salt at household level are also important factors [36].

The level of subclinical iodine deficiency reported here raises policy concerns for effective interventions. This study could serve the policy on salt iodization to monitor progress and implementation. The health care workers can use the findings to initiate appropriate interventions during prenatal services and to counsel clients to improve their iodized salt and milk intake. Based on international criteria for intervention [37], the findings of the present study strongly indicate the need of iodine supplementation to the pregnant women in the area.

## 5. Conclusion

There was moderate to mild iodine deficiency among the pregnant women in the study area. The poor iodine nutritional status of the women resulted from low iodine intake and this in turn was caused by the scarcity of iodized salt utilization in this community. Therefore, there is an urgent need for iodine supplementation to the pregnant women in the setting. Enhancing access to iodized salt and the promotion of its health benefits are priority interventions that need serious attention. Interventions should also focus on young women, on women with no education, and on those with first time pregnancy. Encouraging milk consumption and improving household livestock raring, with particular emphasis to milk cows is also vital.

## Figures and Tables

**Table 1:** Characteristics of pregnant women in Haramaya, eastern Ethiopia, April, 2012.

Variable	Total	No iodine deficiency	Iodine deficiency	*P* value*
	Frequency (%)	Frequency (%)	Frequency (%)	
Age group				0.100
18–24 years	126 (28.97)	18 (14.29)	108 (85.71)	
25–34 years	241 (55.40)	39 (16.18)	202 (83.82)	
35–49 years	68 (15.63)	18 (26.47)	50 (73.53)	
Educational status				0.213
Literate	31 (7.13)	8 (25.81)	23 (74.19)	
Illiterate	404 (92.87)	67 (16.58)	337 (83.42)	
Family size				0.636
<5 persons	41 (18.06)	41 (18.06)	186 (81.94)	
≥5 persons	34 (16.35)	34 (16.35)	174 (83.65)	
Previous pregnancies				0.281
0	87 (20.00)	12 (13.79)	75 (86.21)	
1–3	280 (64.37)	47 (16.79)	233 (83.21)	
≥4	68 (15.63)	16 (23.53)	52 (76.47)	
Pregnancy trimester				0.694
First	52 (11.95)	9 (17.31)	43 (82.69)	
Second	169 (38.85)	26 (15.38)	143 (84.62)	
Third	214 (49.20)	40 (18.69)	174 (81.31)	
Possessed milk cows				0.189
No cow	171 (39.31)	26 (15.20)	145 (84.80)	
One cow	124 (28.51)	28 (22.58)	96 (77.42)	
Two or more cows	140 (32.18)	21 (15.00)	119 (85.00)	
Prenatal visit				0.440
No	112 (25.75)	22 (19.64)	90 (80.36)	
Yes	323 (74.25)	53 (16.41)	270 (83.59)	
Cabbage consumption				0.019
<1 serving/week	334 (76.78)	65 (19.46)	269 (80.54)	
≥1 serving/week	101 (23.22)	10 (9.90)	91 (90.10)	
Household salt				<0.0001
Not iodized	406 (93.33)	62 (15.27)	344 (84.73)	
Iodized	29 (6.67)	13 (44.83)	16 (55.17)	
Consumption of milk				0.500
<3 times/month	230 (52.87)	37 (16.09)	193 (83.91)	
≥3 times/month	205 (47.13)	38 (18.54)	167 (81.46)	

*P values are based on likelihood ratio chi-square test.

**Table 2:** Distribution of median urinary iodine concentration of pregnant women with selected variables, Haramaya, April 2012.

Variable	Percent	Median (*µ*g/L)	Q1 (*µ*g/L)	Q3 (*µ*g/L)	*P* value
Age groups					0.355**
18–24 years	28.97	54.47	19.30	97.97	
25–34 years	55.40	68.66	25.56	151.89	
35 years or older	15.63	68.66	25.56	151.89	
Education					0.776*
Literate	7.13	64.13	23.79	150.75	
Illiterate	92.87	57.80	20.47	109.68	
Number of births					0.168**
0	20.00	49.37	18.84	96.79	
1–3	64.37	56.78	20.34	111.84	
>4	15.63	68.89	31.84	138.72	
Trimester					0.743**
First	11.95	61.87	24.84	114.25	
Second	38.85	58.07	27.33	94.00	
Third	49.20	55.91	13.86	113.79	
Prenatal visit					0.348*
No	25.75	42.84	18.99	124.32	
Yes	74.25	58.78	23.27	110.87	
Milk cows					0.971**
0	39.31	58.34	21.70	103.04	
1	28.51	54.20	20.92	126.87	
2+	32.18	57.95	19.47	113.18	
Milk intake					0.023*
<3 times/month	52.87	47.76	15.70	104.22	
≥3 times/month	47.13	69.14	27.11	113.98	
Cabbage use					0.051*
<1/week	76.78	58.42	23.35	114.28	
≥1/week	23.22	52.10	8.23	89.67	
Household salt					0.018*
Not iodized	93.33	56.57	20.37	103.56	
Iodized	6.67	114.08	28.40	195.64	
Family size					0.669*
<5 persons	52.18	58.07	19.45	103.31	
≥5 persons	47.82	57.93	23.46	113.45	

*P values are based on Mann-Whitney *U* test; **P values are based on Kruskal-Wallis test; Q1: first quartile (25th percentile); Q3: third quartile (75th percentile).

**Table 3:** Factors associated with subclinical iodine deficiency in logistic regression analysis among pregnant women, Haramaya, April 2012.

Variable	Crude OR (95% CI)	*P* value*	Adjusted OR (95% CI)^†^	*P* value*
Age group				
18–24 years	1		1	
25–34 years	0.86 (0.47, 1.58)	0.634	0.59 (0.29, 1.19)	0.138
35–49 years	0.46 (0.22, 0.96)	0.040	0.30 (0.12, 0.75)	0.010
Educational status				
Literate	1		1	
Illiterate	1.75 (0.75, 4.08)	0.195	3.52 (1.30, 9.55)	0.013
Family size				
<5 persons	1		1	
≥5 persons	1.13 (0.68, 1.86)	0.636	3.22 (1.57, 6.58)	0.001
Previous pregnancies				
0	1		1	
1–3	0.79 (0.40, 1.57)	0.508	0.36 (0.15, 0.82)	0.016
≥4	0.52 (0.23, 1.19)	0.122	0.23 (0.07, 0.74)	0.013
Trimester of pregnancy				
First	1		1	
Second	1.15 (0.50, 2.64)	0.740	0.97 (0.38, 2.46)	0.942
Third	0.91 (0.41, 2.02)	0.817	0.78 (0.32, 1.89)	0.582
Possessed milk cows				
No cow	1		1	
One cow	0.61 (0.34, 1.11)	0.108	0.41 (0.21, 0.81)	0.010
Two or more cows	1.02 (0.54, 1.90)	0.960	0.78 (0.38, 1.61)	0.503
Prenatal visit				
No	1		1	
Yes	1.25 (0.72, 2.16)	0.436	1.85 (0.92, 3.70)	0.083
Cabbage consumption				
<3 times/month	1		1	
≥3 times/month	2.20 (1.08, 4.46)	0.029	3.04 (1.37, 6.77)	0.006
Household salt				
Not iodized	1		1	
Iodized	0.22 (0.10, 0.48)	<0.001	0.13 (0.05, 0.32)	<0.0001
Consumption of milk				
<3 times/month	1		1	
≥3 times/month	0.84 (0.51, 1.39)	0.500	0.50 (0.27, 0.93)	0.029

*P values are based on likelihood chi-square test; ^†^adjusted for all variables shown in the table; OR: odds ratio; CI: confidence interval.

## Abbreviations

AOR: Adjusted odds ratio
CI: Confidence interval
EDHS: Ethiopian Health and Demographic Survey
EHNRI: Ethiopian Health and Nutrition Research Institute
ICCIDD: International Council for Control of Iodine Deficiency Disorders
IDD: Iodine deficiency disorders
UIC: Urinary iodine concentration
UNICEF: United Nations Children's Fund
USI: Universal salt iodization
WHO: World Health Organization.
